# Fluorescence spectroscopy and microscopy as tools for monitoring redox transformations of uranium in biological systems†
†Electronic supplementary information (ESI) available: Full experimental details. Luminescence decays and fits, PXRD patterns, scintillation experiment results, additional experimental details and results for control experiments including comparative data with *Escherichia coli*. Anomalous data points resulting from cosmic rays were removed from steady state spectra obtained, and unaltered spectra are included in the ESI. The histogram showing the distribution of lifetime measurements in Fig. 3 shows only pixels with greater than 1000 counts, the full distribution can be found in the ESI. See DOI: 10.1039/c5sc00661a



**DOI:** 10.1039/c5sc00661a

**Published:** 2015-06-09

**Authors:** Debbie L. Jones, Michael B. Andrews, Adam N. Swinburne, Stanley W. Botchway, Andrew D. Ward, Jonathan R. Lloyd, Louise S. Natrajan

**Affiliations:** a School of Chemistry , The University of Manchester , Oxford Road , Manchester , M13 9PL , UK . Email: louise.natrajan@manchester.ac.uk ; Tel: +44 (0)1612751426; b Central Laser Facility , Research Complex at Harwell , Rutherford Appleton Laboratory , OX11 0QX , UK; c School of Earth , Atmospheric and Environmental Sciences , The University of Manchester Oxford Road , M13 9PL , UK; d The Photon Science Institute , The University of Manchester , Oxford Road , Manchester , M13 9PL , UK

## Abstract

We report a study of redox reactions of uranium in model conditions using luminescence spectroscopy, which with its ease and wide availability has the potential to offer new insights into a bioremediation strategy of particular interest – the enzymatic reduction of U^VI^O_2_^2+^ by bacteria such as *Geobacter sulfurreducens*. The inherent luminescent properties of U^VI^O_2_^2+^ have been combined with confocal fluorescence microscopy techniques and lifetime image mapping to report directly on uranium concentration, localisation and oxidation state in cellular systems during uranium bioreduction, suggesting that localisation of uranyl species on the cell membrane surface plays an important role and that extracellular biogenic features form alongside uranyl sorbed cellular species during early stages of the bioreduction. The use of confocal microscopy in tandem with lifetime image mapping offers both improved temporal and spatial resolution (nanoseconds to microseconds and sub-micron respectively) than more conventional X-ray based techniques and offers the potential to image redox reactions occurring *in situ*. Together, these techniques provide an excellent and sensitive probe to assess the coordination environment of uranium during bioreduction processes that are currently being considered for remediation strategies of redox active radionuclides present in contaminated land.

## Abstract 2

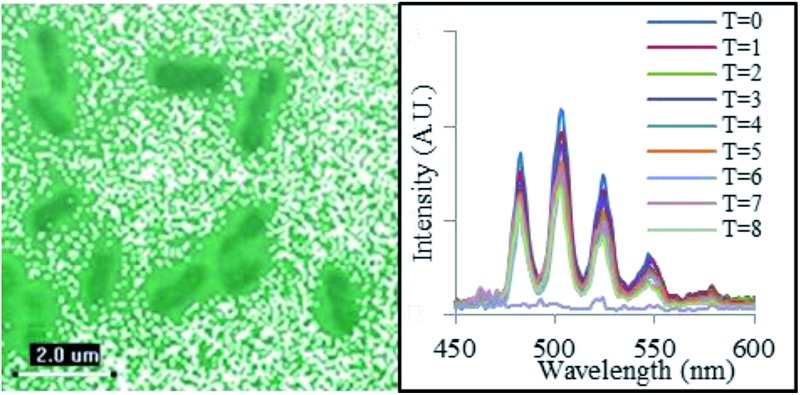
Luminescence spectroscopy, microscopy and lifetime image mapping offers new insights into the bioreduction of *Geobacter sulfurreducens* with uranyl.

## Introduction

The oxidation state of any metal is of vital importance when considering their impact on biological and environmental systems. Oxidation state determines the coordination geometry, bond strength, and Lewis acidity (and therefore the tendency to undergo oligomerisation) and underpins speciation of the metal ion. Additionally, many metal ions are involved in oxidative stress, which arises from the formation of reactive oxygen or nitrogen species and has been implicated in a wide range of diseases.^1^ Optical spectroscopy provides a convenient, non-destructive, and direct method of monitoring the electronic structure of metal ions in complex systems. Luminescence spectroscopy in particular combines high sensitivity, broad applicability, and low cost, making it an attractive option for studying metal oxidation states over more technologically demanding and/or restricted techniques such as X-ray diffraction, X-ray absorption and electron paramagnetic resonance techniques (XRD, XAS, EPR) which often require extensive sample preparation. It has also been combined with optical microscopy to form fluorescence microscopy, confocal microscopy, and, more recently, two-photon excitation microscopy^2^ and super resolution microscopy,^3^ which can provide both spatial and temporal data on a variety of chemical species in a biological setting.^4,5^

Luminescence spectroscopy is an ideal technique for the study of uranium speciation. Since the development of nuclear power and weapons, containment breaches at all stages of the fuel cycle have led to elevated levels of uranium in the environment.^6^ Although there are concerns associated with its radioactivity and long half-life, the hazards of uranium are primarily due to its chemical toxicity.^7^ The dominant form of uranium under oxic, environmental conditions is U^VI^O_2_^2+^, a potent nephrotoxin. The uranyl cation is also very soluble and hence mobile in groundwater and biological systems, therefore remediation techniques focus largely on the reduction of U^VI^O_2_^2+^ to the less soluble U^IV^ cation.^8^ The inherent photophysical properties of the uranyl cation, arising from partially forbidden charge transfer transitions from oxo-based molecular orbitals to non-bonding, unoccupied f-orbitals,^9,10^ provide a convenient means of monitoring uranyl concentration, speciation, and movement without the need of additional imaging agents (such as dye probes). Despite recent time-resolved laser fluorescence spectroscopy (TRLFS) studies into the bioaccumulation of uranium,^11^ the use of optical techniques has been largely underutilised in favour of assay-based and scintillation techniques, to provide concentration data, and X-ray-based techniques such as EXAFS and EDX (coupled with electron microscopy), to provide structural data.

Here, we report the use of luminescence spectroscopy in combination with confocal fluorescence and phosphorescence microscopy and lifetime image mapping in the study of a process of great interest to the remediation of uranium in the environment – the bioreduction of U^VI^O_2_^2+^ to insoluble U^IV^-based mineral-type structures by endogenous bacterial populations.

## Results and discussion

### Luminescence spectroscopy

The bacterium chosen for this study, *Geobacter sulfurreducens*, is a Gram negative bacterium that is ubiquitous in subsurface soils. *Geobacter sulfurreducens* is well known to enzymatically reduce U^VI^O_2_^2+^ under anaerobic conditions^12–15^ and was grown according to literature precedent.^16^ This reduction process has previously been studied by assay-based techniques to yield concentration data^12^ and EXAFS to give structural data,^14^ however many questions still remain on the exact enzymatic mechanisms responsible for the reduction process and despite its sensitivity and greater spatial resolution, luminescence spectroscopy remains underutilised in this field. Under the conditions required for bioreduction to occur (30 mM NaHCO_3_, 5 mM UO_2_(CH_3_CO_2_)_2_, see ESI† for further details) in the absence of bacterial cells, excitation at 420 nm resulted in characteristic uranyl emission centred at 525 nm. Strong coupling of the electronic energy levels with the Raman active symmetric O–U–O stretching mode often results in a vibronically resolved spectrum with several distinct emission bands between 450 and 650 nm. The spectra observed here show a single broad peak consistent with emission from uranyl at near-neutral and basic conditions.^17–19^ The luminescence lifetime could not be modelled adequately as a single exponential decay (Table 1), and instead was fitted to a biexponential decay with two components of 2.2 μs and 7.1 μs, each contributing approximately equally. Thermodynamic modelling carried out using the PHREEQC^20^ software package, suggests that under these conditions, uranium exists primarily as carbonate complexes UO_2_(CO_3_)_3_^4–^ (83%), UO_2_(CO_3_)_2_^2–^ (8%), and (UO_2_)_3_(CO_3_)_6_^6–^ (3%). As most uranyl carbonate complexes are known to be non-emissive at room temperature,^21^ it is likely that minor oligomeric and uranyl hydroxide species contribute disproportionately to the emissive properties of the system.

**Table 1:** Collected lifetime data from various solutions, percentages indicate contribution to biexponential decay model

Solution	Temp/K	Emission lifetime/μs (%)
5 mM UO_2_(CH_3_CO_2_)_2_, 30 mM NaHCO_3_	293	2.21 ± 0.13 (51)
		7.06 ± 0.31 (49)
5 mM UO_2_(CH_3_CO_2_)_2_, 30 mM NaHCO_3_, *Geobacter sulfurreducens*	293	8.84 ± 0.46 (61)
		20.98 ± 1.17 (39)
5 mM UO_2_(CH_3_CO_2_)_2_, 30 mM NaHCO_3_	77	1198.20 ± 9.02
5 mM UO_2_(CH_3_CO_2_)_2_, 30 mM NaHCO_3_, *Geobacter sulfurreducens*	77	1125.62 ± 12.63

In the presence of metabolically active washed cell suspensions of *Geobacter sulfurreducens* a change in the emissive properties was observed (Fig. S1†), with the steady-state emission spectrum showing subtle changes in profile significant increases in intensity the luminescence lifetime to 8.8 (61%) and 21 μs (39%). As aliquots taken from a bacterial cell suspension in the absence of uranium show little autofluorescence or scattering (ESI, Fig. S1†) these results represent a change in uranyl speciation in the presence of bacteria, suggesting that either the uranyl is sorbing onto the surface of the cells, being taken up into the cells, or that the bacteria are releasing biogenic complexants. Indeed, efficient biosorption of uranium onto living and dead cellular material and the complexation of uranyl species by biogenic carbonate and phosphate are well-known phenomena,^6^ however, interestingly, these have not previously been implicated as a step in the bioreduction of U^VI^.

**Fig. 1:**
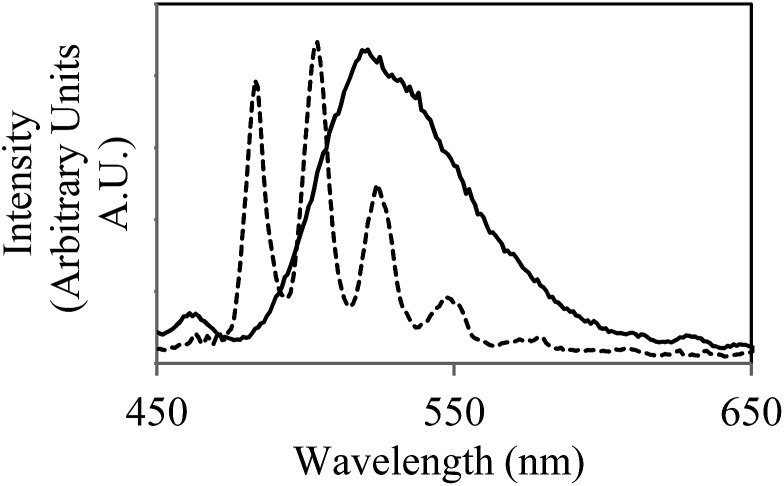
Emission spectra of the uranyl cation in a carbonate buffer solution (30 mmol) in the presence of *Geobacter sulfurreducens* at room temperature (solid line) and 77 K (dotted line), (*λ*_ex_ = 420 nm). Aliquot obtained immediately after introduction of UO_2_(CH_3_CO_2_)_2_ (5 mmol) to microcosm and brief agitation.

In a frozen solution at 77 K, the corresponding emission spectrum was significantly more well-resolved (Fig. 1), with the radiative decay now exhibiting monoexponential kinetics and a determined lifetime of 1125 μs. This suggests a shift in speciation toward a single emissive complex. It appears that if the interaction of U^VI^O_2_^2+^ with the cells is maintained at this temperature it does not significantly contribute to the fluorescent properties of the system. Indeed, at this temperature, the structural integrity of the cellular structure of the bacteria is likely to be compromised. A control study with a uranyl nitrate solution shows the same steady-state spectrum and emission lifetime (ESI†), demonstrating that the emissive species in the presence of *Geobacter sulfurreducens* is not a uranyl acetate complex. Uranyl carbonate species are known to be emissive under cryogenic conditions,^21,22^ showing significant variation in both the energy of the vibronic bands and the emission lifetimes. Comparison of the peak values and lifetime measurements with literature reports for uranyl carbonate and hydroxide species^21–23^ did not allow identification of the emissive species in this system; it does however suggest that if multiple species were present, this would be visible in both the steady-state and time-resolved spectra. The simplified speciation enabled the concentration of uranyl to be monitored and quantified over the course of the bioreduction experiment (see ESI† for calibration experiments). At regular time points an aliquot of the solution was removed from the reaction and frozen in liquid nitrogen, an emission spectrum was then obtained under a standardised instrumental set-up (see ESI† for further details). Over eight hours the emission intensity showed a general decrease, and after one day the solution was completely non-emissive, suggesting that microbial reduction had proceeded to completion. This interpretation was supported by scintillation techniques, (ESI†) and the formation of the U^IV^ compound uraninite (UO_2_), the primary product of microbial reduction of U^VI^ with *Geobacter sulfurreducens*,^24–26^ confirmed by powder X-ray diffraction (Fig. S12, ESI†). Control studies carried on dead cells (ESI, Fig. S14†) showed only a slight decrease in fluorescence intensity over the course of 24 hours. This is in good agreement with literature reports and demonstrates that the process is enzymatic and not simply dependant on biogenic reductants which may be released in the absence of metabolic activity. It also demonstrates that the observed decrease in fluorescence intensity is unlikely to be simply be due to sorption of uranium onto cellular material, as the extent of sorption by dead cellular material is known to be generally equal to or greater than the extent of sorption by living biomass.^27,28^

**Fig. 2:**
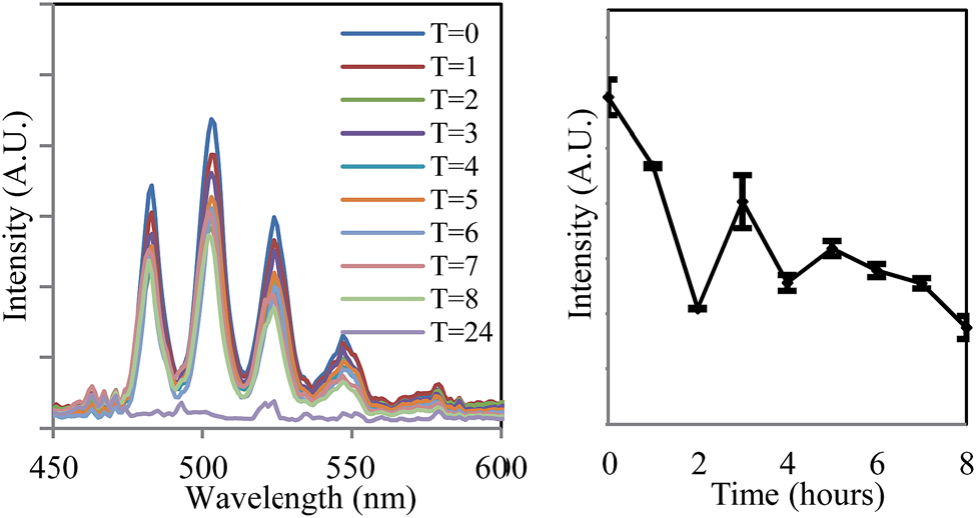
The decrease in the intensity of uranyl emission over time in the steady-state emission spectrum of anaerobic uranyl solutions containing *Geobacter sulfurreducens* (*λ*_ex_ = 420 nm, 77 K).

The ease and speed with which photoluminescence spectroscopy can be carried out allowed this process to be studied at higher temporal resolution than previously available,^14^ revealing an unusual feature during the early stages of the bioreduction. Although the emission intensity of U^VI^O_2_^2+^ underwent a general decrease over eight hours, the rate of this decline was not consistent (Fig. 2, right). Instead, the process was seen to occur in several stages, in which an initial sharp decrease in emission intensity was followed by partial increase in intensity. This distinctive ‘saw tooth’ pattern was obtained reproducibly and cannot be attributed to instrumental or experimental error. There are several possible processes that may account for this phenomenon. Although the reoxidation of U^VI^O_2_^2+^ to U^VI^ has been observed under nominally reducing conditions, this process occurs over much greater timescales (up to 500 days) and in more ‘geomimetic’ conditions in which nitrate, Fe^III^ and Mn^IV^ may act as terminal electron acceptors.^29,30^ An alternative explanation may rest in changes in the uranyl speciation. If the most emissive U^VI^O_2_^2+^ species was preferentially reduced by the bacteria^31,32^ a sharp decrease in emission intensity resulting from bioreduction may be followed by a gradual increase as the system re-equilibrates. This explanation, however, does not take into account the simplified uranium speciation profile at 77 K at which these results were obtained. A more likely explanation, it seems, is that the delayed rise in fluorescence intensity is due to the disproportionation of an unstable U^V^O_2_^+^ intermediate which is non fluorescent in the 450–600 nm spectral window employed. As enzymatic U^VI^O_2_^2+^ reduction, a first order reaction with regards to [U^VI^O_2_^2+^] occurs meaning the concentration of U^VI^O_2_^2+^ and rate of reaction steadily decrease. At the same time, the concentration of U^V^O_2_^+^ increases and the rate of disproportionation, a second-order reaction, will increase quadratically. This may account for the fluctuations in the concentration, and hence emission intensity, of U^VI^O_2_^2+^ as it is gradually reduced to U^IV^ *via* an unstable U^V^O_2_^+^ intermediate. Moreover, liquid scintillation counting experiments over the same time period show a linear decrease in total soluble uranium content, giving further weight to this argument (ESI, Fig. S13†). Indeed, direct and indirect evidence has previously pointed to the existence of a U^V^O_2_^+^ intermediate,^9^ suggesting that the enzymatic reduction of U^VI^O_2_^2+^ by *Geobacter sulfurreducens* is in fact a one electron reduction, followed by disproportionation of U^V^O_2_^+^ to form U^IV^ and U^VI^O_2_^2+^, and it appears that emission spectroscopy supports this conclusion.

### Fluorescence microscopy

Having shown that the inherent emission of the U^VI^O_2_^2+^ ion provides a suitable handle for monitoring uranium oxidation state during enzymatic reduction by *Geobacter sulfurreducens*, the same process was then studied by fluorescence microscopy at sub-micron spatial resolution in order to probe the locality/(bio) distribution of uranyl both within bacterial communities and with isolated single bacterium. The bioreduction reaction was carried out as described previously, with the only exception being that aliquots were placed directly onto a glass slide for observation by microscopy, which was carried out at room temperature. The microscope was equipped with a 405 nm diode laser; while this is not the peak excitation wavelength it does excite into the same uranyl absorption band as used in the spectroscopic experiments. Additionally a CCD-based spectrometer enabled the collection of steady-state spectra on objects observed under the microscope, full instrumental details can be found in the ESI.† Immediately after the introduction of bacteria to the uranyl solution, relatively large extracellular masses (*ca.* 7–15 microns in size) that displayed intense fluorescence in the green-channel were clearly visible alongside the bacterial cells.

**Fig. 3:**
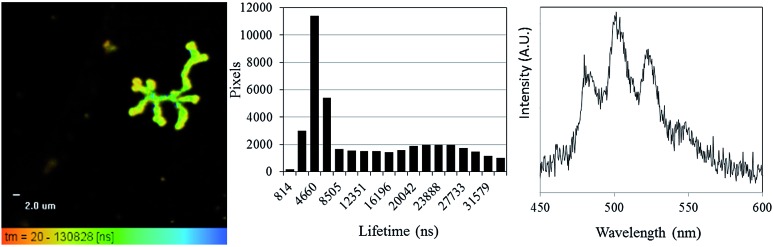
Phosphorescence lifetime image map focussing on a representative extracellular feature in samples taken immediately after the introduction of *Geobacter sulfurreducens* to uranyl acetate solution (left), a histogram showing the distribution of lifetime measurements (centre), and a representative, uranyl spectrum from one such extracellular feature (right) (*λ*_ex_ = 405 nm, room temperature).

Fluorescence lifetime image mapping (FLIM) and phosphorescence lifetime image mapping (PLIM) revealed that these features displayed long-lived emission, up to 130 μs, (Fig. 3, left) suggesting that this was due to uranyl fluorescence rather than biological autofluorescence. A steady-state spectrum obtained directly from one such extracellular mass confirmed this, showing characteristic, well-resolved uranyl emission (Fig. 3, right) along with weak autofluorescence/background emission at longer wavelengths (ESI, Fig. S6†). Since a wide range of phosphorescent lifetimes were observed (0.8 to 130 μs), these features cannot be a single, homogeneous material such as re-precipitated uranyl acetate, but are more likely to be U^VI^O_2_^2+^ species that have been sorbed to or complexed by biological material or precipitated out as amorphous or polycrystalline acetate, carbonate or hydroxide salts. This also supports the results of the spectroscopic experiments and suggests that the change in emission lifetime observed in the bulk solutions may be due to coordination of U^VI^O_2_^2+^ to complexants released by the bacterial cultures. In samples prepared several hours after the introduction of uranium these features were no longer visible. Instead, the bright field images showed the *Geobacter sulfurreducens* cells surrounded by nanoparticulate matter that displayed no fluorescence or phosphorescence. It appears that as the bioreduction proceeds, and the solution-phase concentration of U^VI^O_2_^2+^ decreases, any precipitated U^VI^O_2_^2+^ was converted, possibly *via* re-dissolution, to the U^IV^ product of the reaction, which formed the insoluble and non-emissive mineral uraninite (UO_2_).^33^

**Fig. 4:**
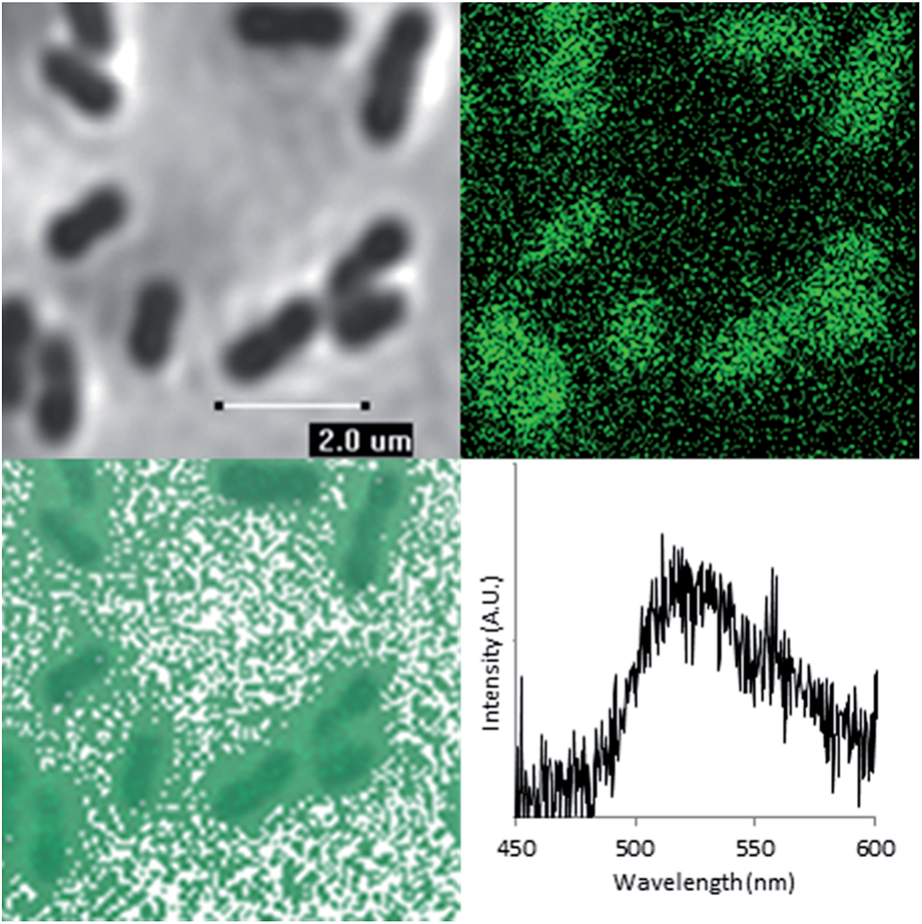
Bright-field microscopy image (top left), FLIM image (top right), Overlaid bright-field and FLIM image (bottom left) of *Geobacter sulfurreducens* and representative spectrum taken from one such bacterium (*λ*_ex_ = 405 nm, room temperature).

Having demonstrated that FLIM and PLIM are suitable methods for studying the bioreduction of uranium by *Geobacter sulfurreducens*, attention turned to the cells themselves. The rod-shaped, micron-sized cells were clearly visible in the bright-field image (Fig. 4, top left) and in the absence of uranium showed very little autofluorescence. When uranyl acetate was added, the cells became clearly visible by FLIM, (Fig. 4, top right) having experienced a significant enhancement in fluorescence. The bright field and FLIM images are clearly superimposable (Fig. 4, bottom left) indicating that the emissive uranium is associated with the bacterial cells; no significant emission was recorded in the PLIM window. As observed in the solution luminescence spectroscopy experiments, the spectrum obtained from the bacteria showed a broad, unresolved peak centred at 515 nm consistent with the presence of multiple emissive species, (Fig. 4, bottom right) along with marginal autofluorescence in the red region (ESI, Fig. S7†). Over several hours this fluorescence diminished, coinciding with the conversion of extracellular, U^VI^O_2_^2+^-containing material and the formation of nanoparticulate UO_2_. In contrast to the extracellular material, the U^VI^O_2_^2+^ fluorescence from the surface of the cells was extremely short-lived (*ca.* 300–800 ps, ESI Fig. S17†). This is not unexpected, as the reduction potential of the uranyl cation is considerably increased on photoexcitation and transitory reduction to a U^V^O_2_^+^ species is considered to be a significant quenching mechanism.^34^ In fact, the short fluorescent lifetime may principally be due to the presence of species involved in the electron-transport chain in which U^VI^O_2_^2+^ acts as the terminal electron acceptor. This is further supported by control experiments carried out on a *Escherichia coli*, which is known to sorb but not reduce U^VI^O_2_^2+^.^35,36^ These studies demonstrated an enhancement in fluorescence intensity of *E. coli* cells in the presence of uranyl acetate but significantly longer fluorescence lifetimes (ESI Fig. S15 and S16†).

Furthermore, altering the excitation focus in the *z* direction (±2 μm), resulted in a slight decrease in emission intensity upon passing through the *Geobacter sulfurreducens* cells. Although the spatial resolution of the FLIM and PLIM images do not enable the precise location of the uranium within the bacterial cells to be determined, these data may indicate that the uranium is principally associated with the cell walls and/or the outer cell membranes rather than incorporated into the cell. However, since the reduction in lifetime was not observed by spectroscopy in the bulk samples on short timescales (ns), it is likely that the surface bound species make up a very minor component of the total uranyl speciation. Further, gene deletion experiments alongside proteomic studies have suggested that both outer membrane and periplasmic c-type cytochromes play a central role in extracellular electron transport in the bioreduction of U^VI^O_2_^2+^ with *Geobacter sulfurreducens*.^38^

## Conclusions

In summary, we have demonstrated the utility of readily available, facile luminescence spectroscopy in studying the bioreduction of U^VI^O_2_^2+^ by *Geobacter sulfurreducens* over a 24 hour time period. The speed at which a spectrum can be obtained, and the minimal sample preparation involved has allowed the progress of the reaction to be studied *in situ*, importantly, at shorter time-increments than previously possible, providing supporting evidence towards the role of a U^V^O_2_^+^ intermediate. We have also demonstrated for the first time the viability of using the inherent fluorescent properties of the U^VI^O_2_^2+^ to report directly on the localisation of uranium by fluorescence and phosphorescence lifetime image mapping without additional stains or imaging agents or the need for sample-altering preparation techniques such as fixation. Interestingly, long-lived phosphorescent extracellular inorganic-organic features are observed in the early stages (first hour) of the bioreduction process by both PLIM and FLIM techniques. Fluorescence microscopy of *Geobacter sulfurreducens* cells shows very little autofluorescence in the absence of uranium, however on the addition of uranyl acetate there is an immediate increase in emission associated with the surface of the cells, this suggests a previously unconsidered step in the enzymatic reduction process – the absorption of U^VI^O_2_^2+^. The extremely short luminescent lifetime suggest that the uranyl is interacting with electron-rich species, potentially electron transfer proteins localised towards the periphery of the outer membrane of the Gram-negative bacterial cell, such as cytochromes (including c-type) which have been implicated in the reduction of uranium^37^ and those that are known to bind U^VI^O_2_^2+^.^38–40^ The identification of extracellular material has suggested the applicability of this technique to the study of other bioremediation techniques such as biosorption, bioaccumulation and biomineralisation. This approach could provide new insights into the fate of uranium in more complex microbial–mineral–ground water systems, which are currently poorly understood. Further work will also aim at incorporating two-photon microscopy as a potential means of providing additional spectroscopy and spatial data.
