# Supplementary material for: Fluorescence spectroscopy and microscopy as tools for monitoring redox transformations of uranium in biological systems
Source: Chem Sci. 2015 Jun 9;6(9):5133–8. doi: 10.1039/c5sc00661a (PMC5666681; doi:10.1039/c5sc00661a)
Supplement: Supplementary file 1 [file SC-006-C5SC00661A-s001.pdf]

Electronic Supplementary Material (ESI) for Chemical Science.
This journal is © The Royal Society of Chemistry 2015

Electronic Supporting Information (ESI) for Fluorescence spectroscopy and microscopy as tool for monitoring redox transformations of uranium in biological systems

Debbie L. Jones, Michael B. Andrews, Adam N. Swinburne, Stanley W. Botchway, Andrew D. Ward, Jonathan R. Lloyd, and Louise S. Natrajan*

Sample Preparation

All manipulations of cells were carried out under an atmosphere of N_2. Late-log-phase cultures were harvested by centrifugation and washed twice in $NaHCO_3$ buffer (30 mM, pH 7, degassed with N_2-CO_2 80:20 mix). Aliquots of the washed cell suspensions were added to a final concentration of 0.5 mg mL^{-1} dry weight biomass, by use of a syringe fitted with a needle, to anaerobic bottles sealed with butyl rubber stoppers containing uranyl(VI) acetate (5mM) in $NaHCO_3$ buffer (30 mM, pH 7) creating the microcosm for the experiment. No electron donor was added as the acetate from the uranyl acetate acts as the electron donor in this case. No further nutrients were added to prevent cell growth from interfering with the experiments.

Samples were removed from the microcosm every hour, by use of a syringe fitted with a needle and filtered into a young's tap cuvette, a low temperature EPR tube or placed onto a slide and sealed with a cover slip, for room temperature luminescence, low temperature luminescence and microscopy studies respectively.

Fluorescence Studies

Steady state emission spectra were recorded on an Edinburgh Instrument FP920 Phosphorescence Lifetime Spectrometer equipped with a 5 watt microsecond pulsed xenon flash lamp and a 450 watt steady state xenon lamp and a red sensitive photomultiplier in peltier (air cooled) housing, (Hamamatsu R928P). Lifetime data were recorded following 420 nm excitation with a 5 Watt xenon flash lamp (Edinburgh Instruments), using multichannel scaling. Lifetimes were obtained by tail fit on the data obtained, and quality of fit judged by minimization of reduced chi-squared and residuals squared.

For consistency and in order to ensure reproducibility steady-state luminescence experiments were carried out using identical instrumental settings and all data reported are an average of at least 3 runs:

Table S1. Instrumental setting used to collect steady-state emission spectra.

Instrument Setting	Value
Step	1.00 nm
Dwell Time	0.2 s
Repeats	5
Excitation Monochromator Bandwidth	2.00 nm
Emission Monochromator Bandwidth	2.00 nm

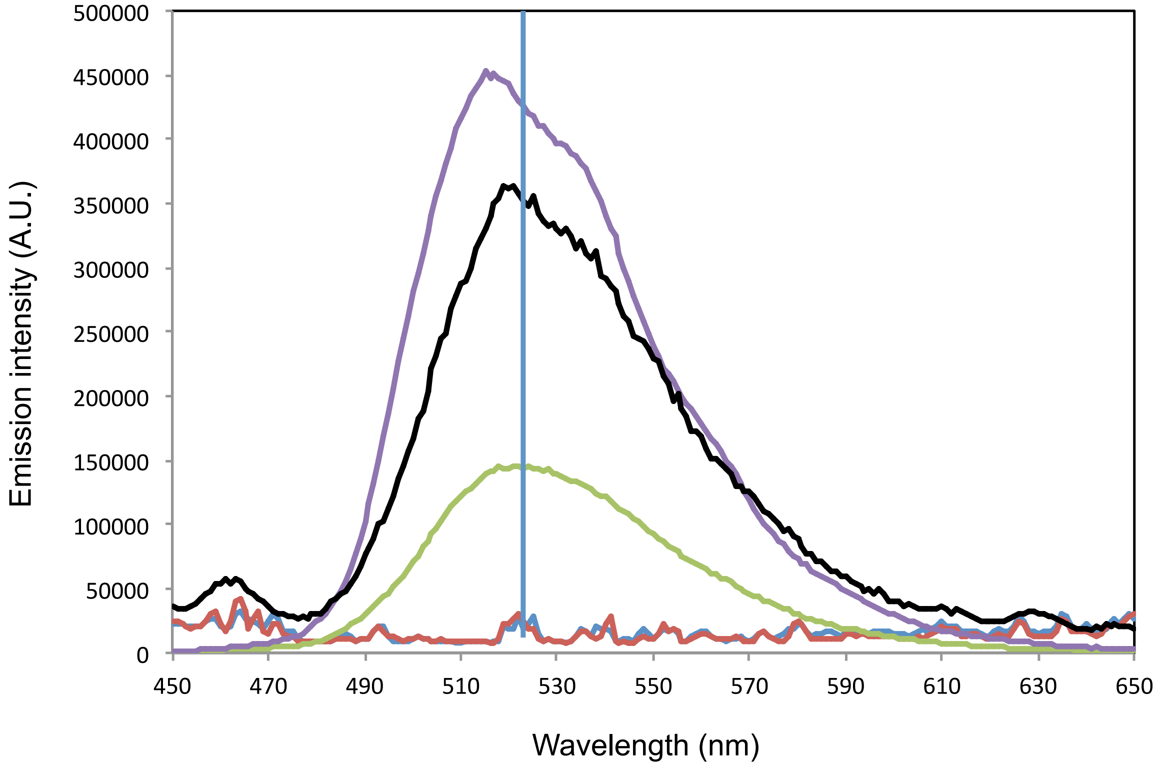

Fig. S1. Steady state emission profile of room temperature solutions of uranyl acetate (5 mmol) in sodium carbonate buffer (30 mmol) in the absence (green line) and presence of *Geobacter sulfurreducens* (purple line) and following freezing and defrosting (black line) as well as the scattering caused by OD600 0.1 *Geobacter sulfurreducens* in carbonate buffer in the absence of uranium (room temperature – red line, 77K blue line). Blue vertical line placed at the emission maximum of the green spectrum.

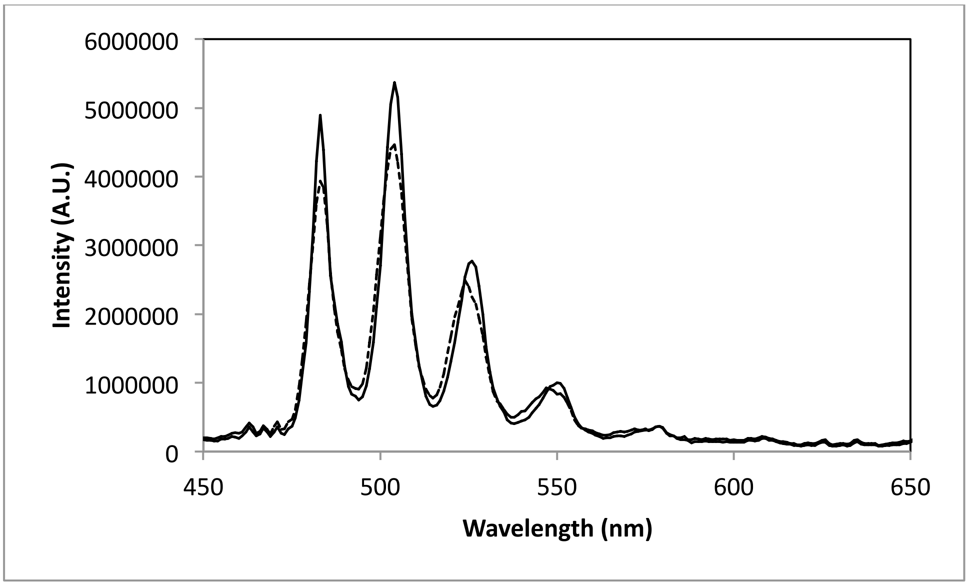

Fig. S2. Steady state emission profile of 77 K solution of uranyl acetate (5 mmol) in sodium carbonate buffer (30 mmol) in the absence (solid line) and presence of *Geobacter sulfurreducens* (dashed line). (λ_{ex} = 420 nm)

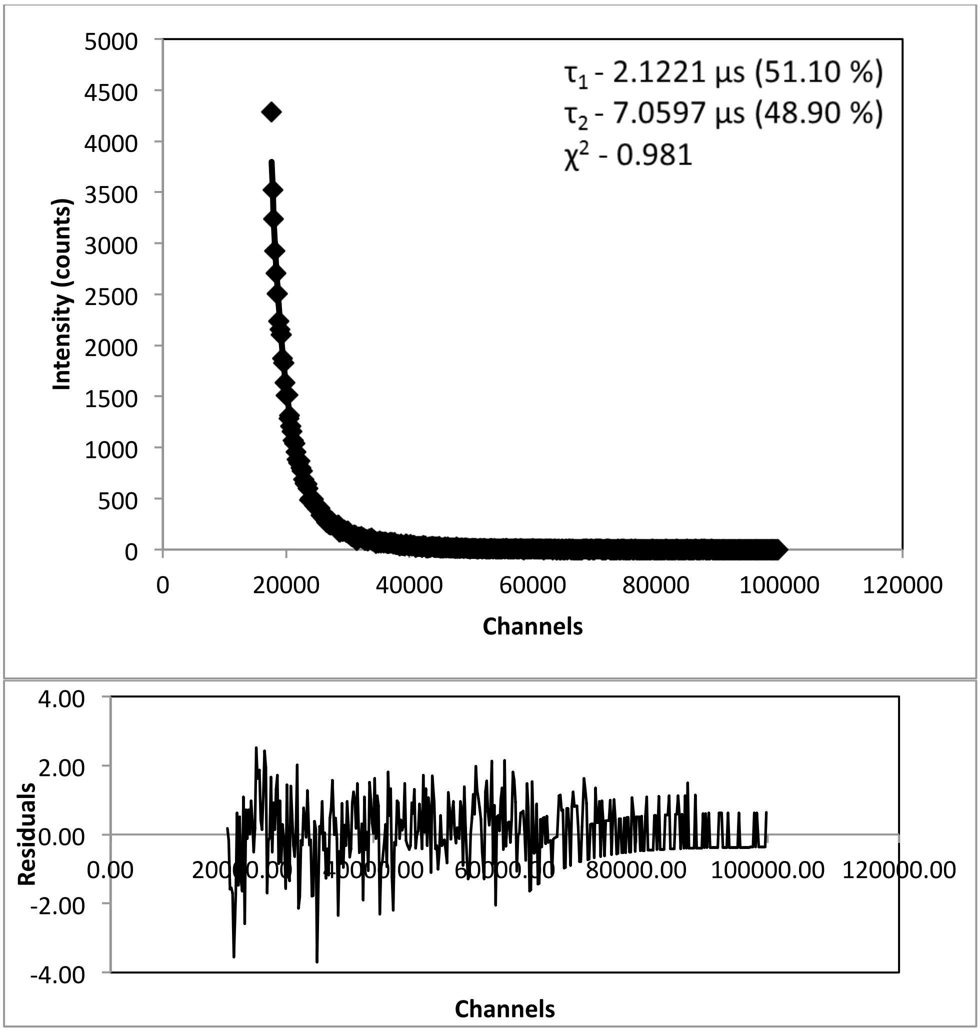

Fig. S3. Kinetic trace of uranyl acetate in buffer solution at room temperature (top); black squares represent recorded data, black line represents polyexponential fit. Residuals of fit also plotted (bottom). (λ_{ex} = 420 nm, λ_{em} = 503 nm)

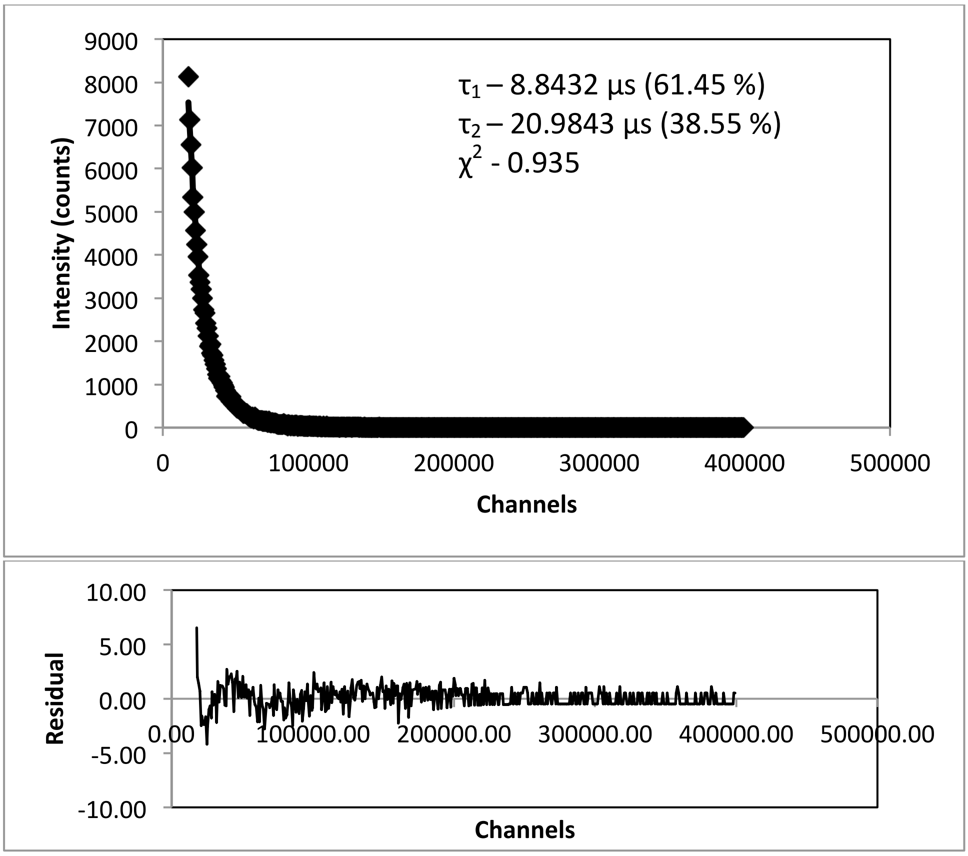

Fig. S4. Luminescent lifetime of uranyl acetate and *Geobacter sulfurreducens* in buffer solution at room temperature (top); Black squares represent recorded data, black line represents polyexponential fit. Residuals of fit also plotted (bottom). (λ_{ex} = 420 nm, λ_{em} = 503 nm)

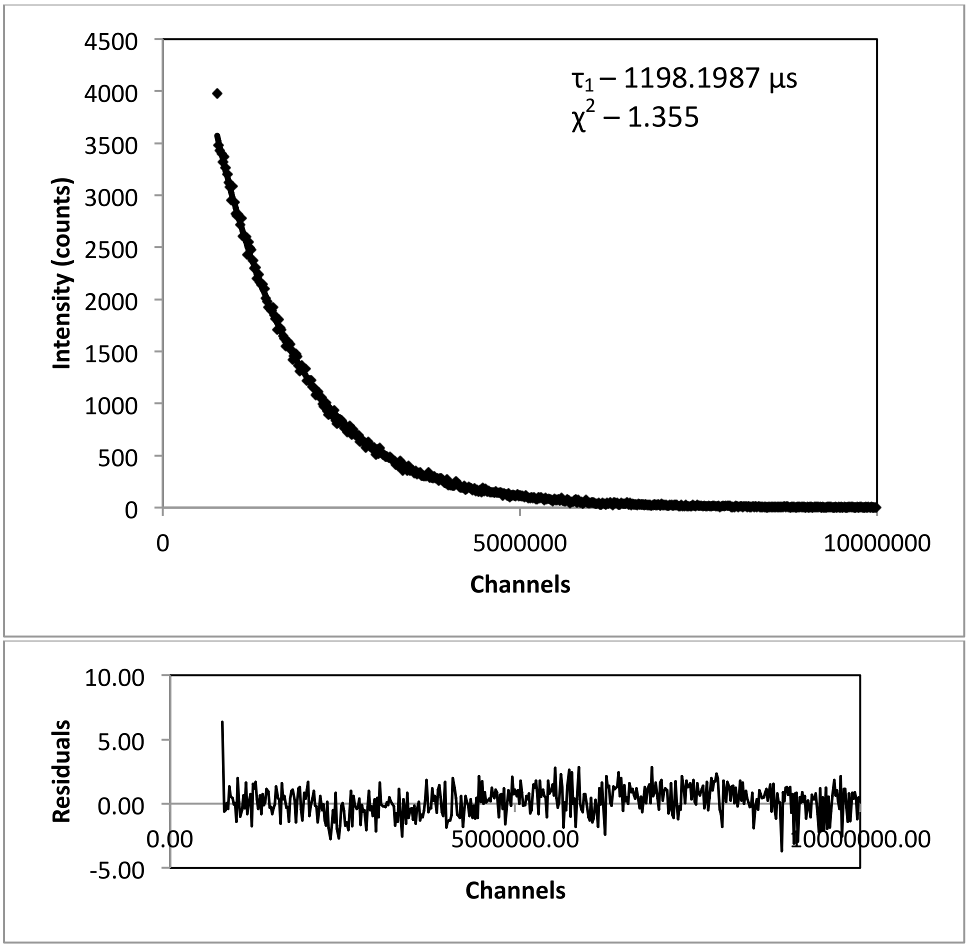

Fig. S5. Luminescent lifetime of uranyl acetate in buffer solution at 77 K (top); black squares represent recorded data, black line represents polyexponential fit. Residuals of fit also plotted (bottom). (λ_{ex} = 420 nm, λ_{em} = 503 nm)

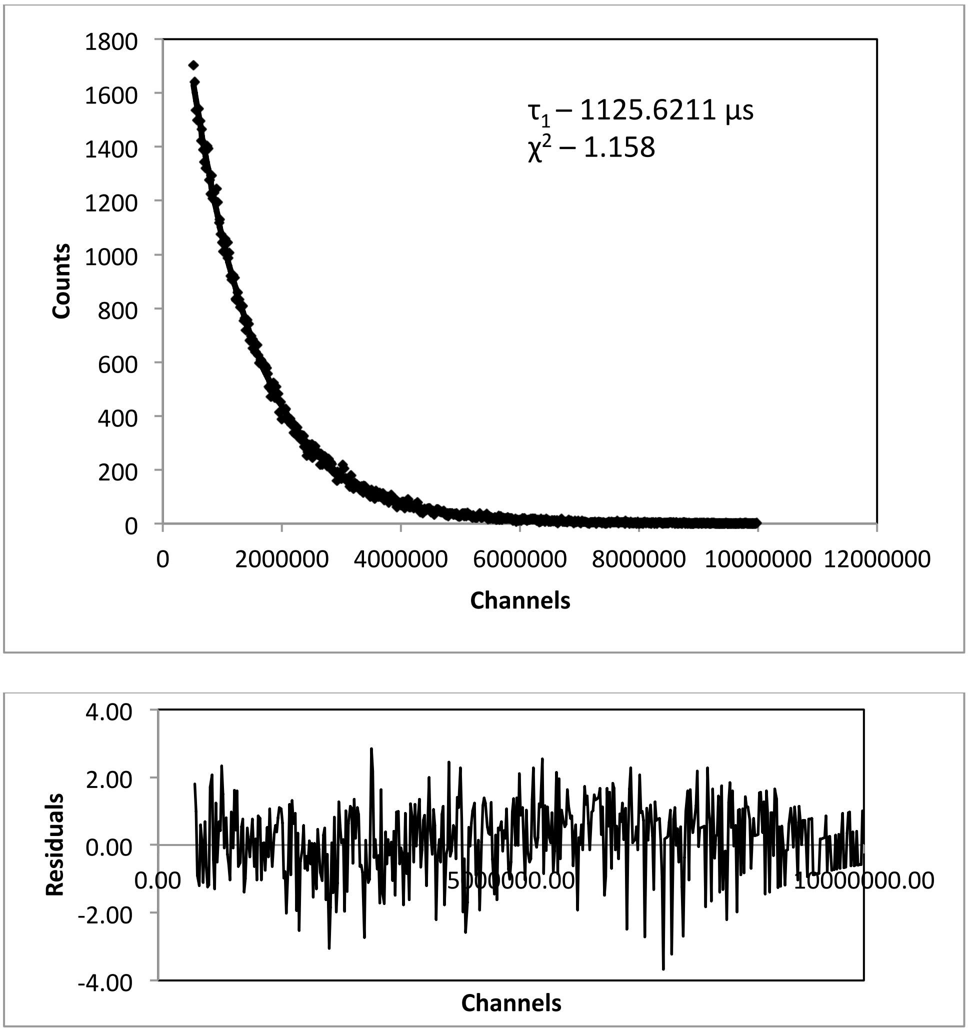

Fig. S6. Luminescent lifetime of uranyl acetate with *Geobacter sulfurreducens* in buffer solution at 77 K (top); black squares represent recorded data, black line represents polyexponential fit. Residuals of fit also plotted (bottom). (λ_{ex} = 420 nm, λ_{em} = 503 nm)

Uranyl nitrate control study

In order to gain further insight into the speciation of uranyl solutions at 77 K a control study was carried out using the same concentration of $UO_2(NO_3)_2$ in carbonate buffer. At 77 K the steady state and luminescence lifetime spectra were identical to those obtained from the uranyl acetate solution.

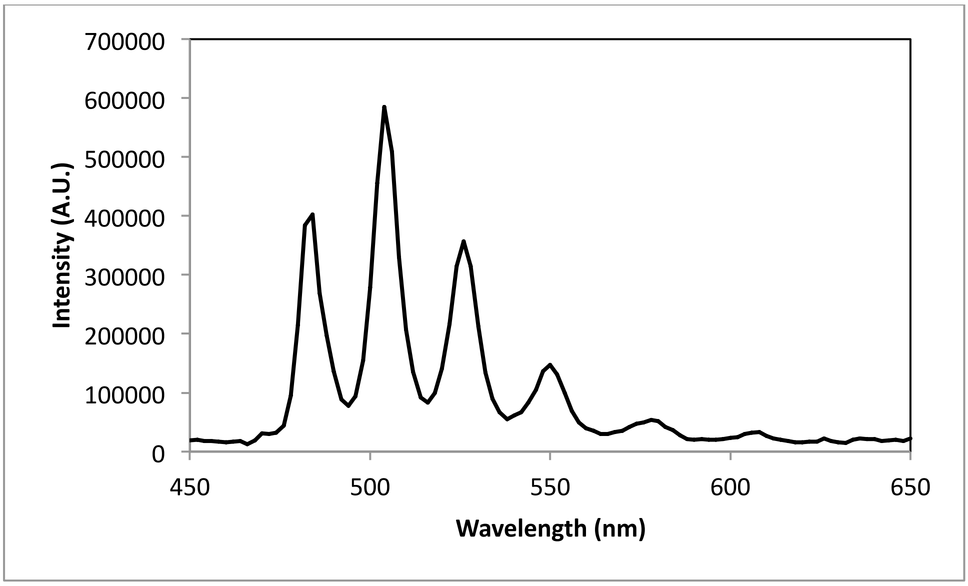

Fig. S7. Steady-state emission spectrum of $UO_2(NO_3)_2.6H_2O$ (5 mM) and $NaHCO_3$ (30 mM) in frozen aqueous solution (77 K). (λ_{ex} = 420 nm)

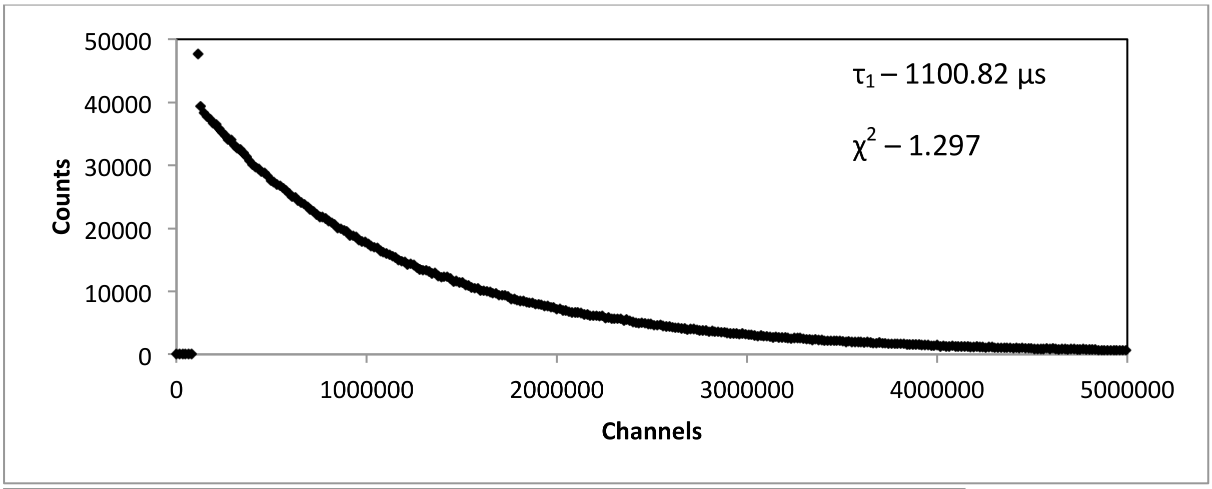

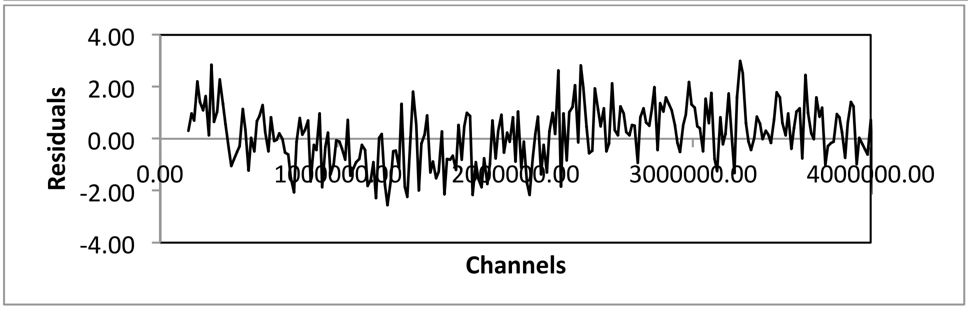

Fig. S8. Luminescent lifetime of uranyl nitrate in buffer solution at 77 K (top); black squares represent recorded data, black line represents polyexponential fit. Residuals of fit also plotted (bottom). (λ_{ex} = 420 nm, λ_{em} = 503 nm)

Uranyl concentration vs. Emission intensity control study

In order to ensure that the emission intensity of the frozen solutions (77 K) represented the uranyl concentration and there were no inner filter effects operative, a calibration study was carried out using varying concentrations of uranyl acetate in the carbonate buffer solution. It was found that there was a linear relationship between uranyl concentration and emission intensity.

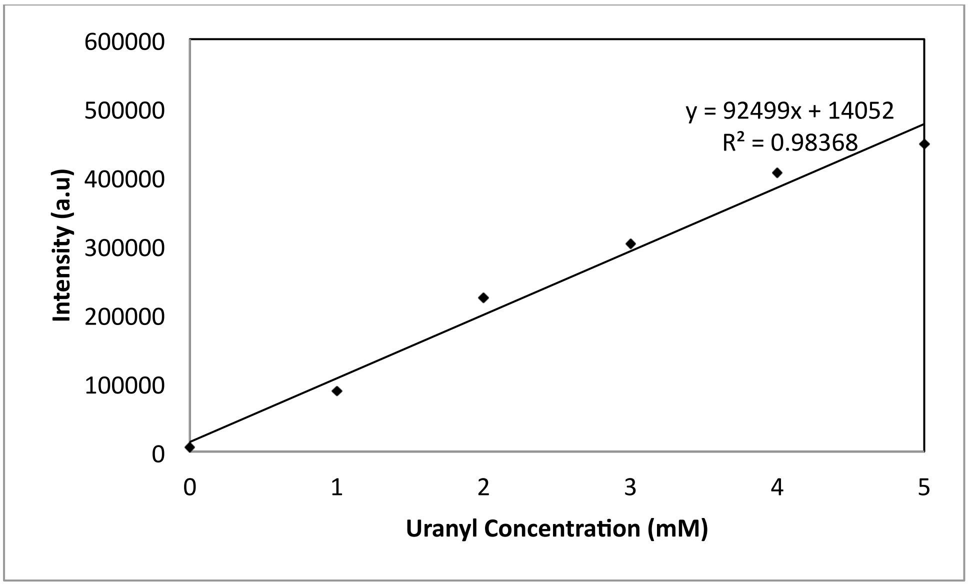

Fig. S9. Luminescence Intensity of the main 503 nm emission band plotted against uranyl concentration in carbonate buffer solution (30 mM $NaHCO_3$). (λ_{ex} = 420 nm, λ_{em} = 503 nm)

Microscopy

The 1 photon-excitation FLIM-PLIM is a commercial system from Becker and Hickl with a 405 nm pulsed laser system. Fluorescence lifetime images were obtained using a modified confocal microscopy apparatus, constructed in the Central Laser Facility, which has a Nikon eC1 scanhead. Laser light was focused through a x60 water immersion objective with an NA of 1.2 on an inverted Nikon microscope (TE2000-U). Emission was collected without descanning, by-passing the scanning system, and passed through a bandpass filter (BG39, Comar). The scan was operated in the normal mode and line, frame and pixel clock signals were generated and synchronized with an external fast micro-channel plate photomultiplier tube (Hamamatsu R3809U) used as the detector. These were linked via a time-correlated single photon counting (TCSPC) PC module SPC830 (Becker and Hickl). The set-up provided instrument quantum efficiencies of more than 50% with single photon detection capabilities. Steady-state grey-scale multiphoton images (8 bit, up to 256 x 256 pixels) are produced by binning all decay photons as a single channel. Emission lifetime images were obtained by analysing the decay at individual pixels using a single or double exponential curve fitting following some modification to the standard Becker-Hickl SPCImage analysis software (B&H SPCImage 2.94) to allow analysis of the microsecond decay domain. A thresholding function within the analysis software ensured that non-correlating photons leading to background noise arriving at the detector were not included in the analysis. The lifetime image data are presented without further image processing. Steady state spectra were recorded using an Ocean Optics USB2000+ spectrometer.

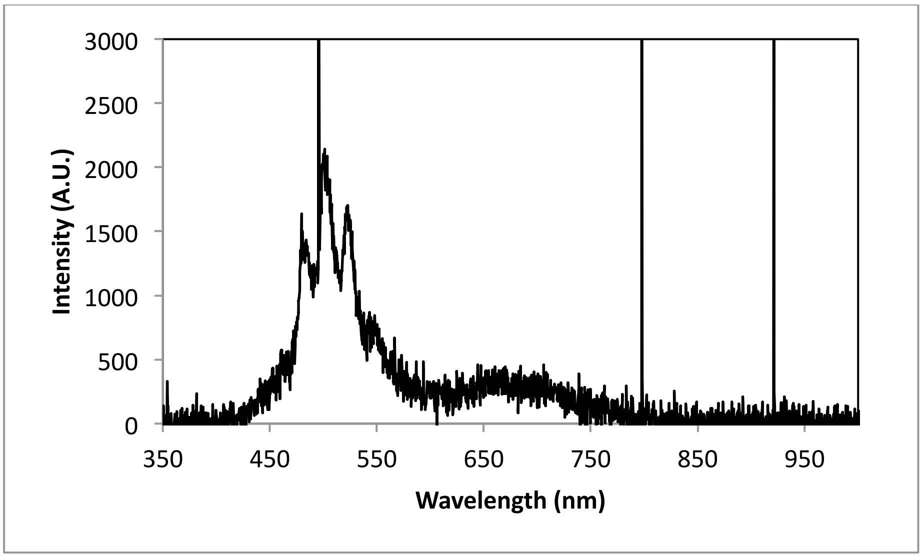

Fig. S10. Example of characteristic uranyl spectrum obtained from extracellular material following excitation at 405 nm. High intensity peaks (full height 5000 – 20000 A.U.) due to interference by cosmic rays and removed from Fig. 3 in main text.

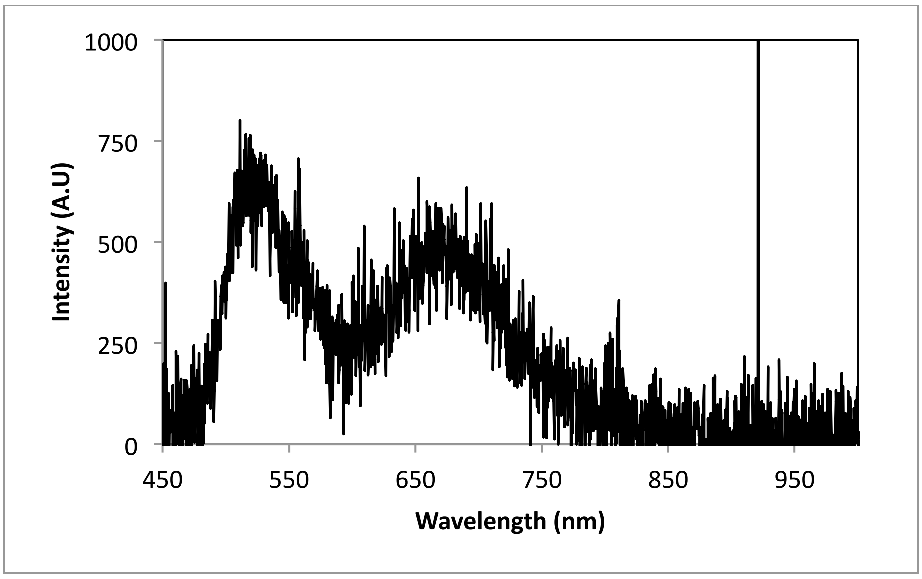

Fig. S11. Example of broad uranyl (centred at 510 nm) and biological autofluorescence (centred at 670 nm) spectrum of *Geobacter sulfurreducens* following excitation at 405 nm. High intensity peak (full height 7800 A.U.) due to interference by cosmic rays.

Powder X-ray Diffraction (PXRD)

To identify the black precipitate produced by the bioreduction experiments an aliquot taken at the end of the experiment was centrifuged to separate insoluble material. This was then dried and applied directly to the sample holder and was analysed via powder X-Ray Diffraction (pXRD; Bruker D8 Advance using Cu-K[illegible] radiation and EVA 14 analytical software).

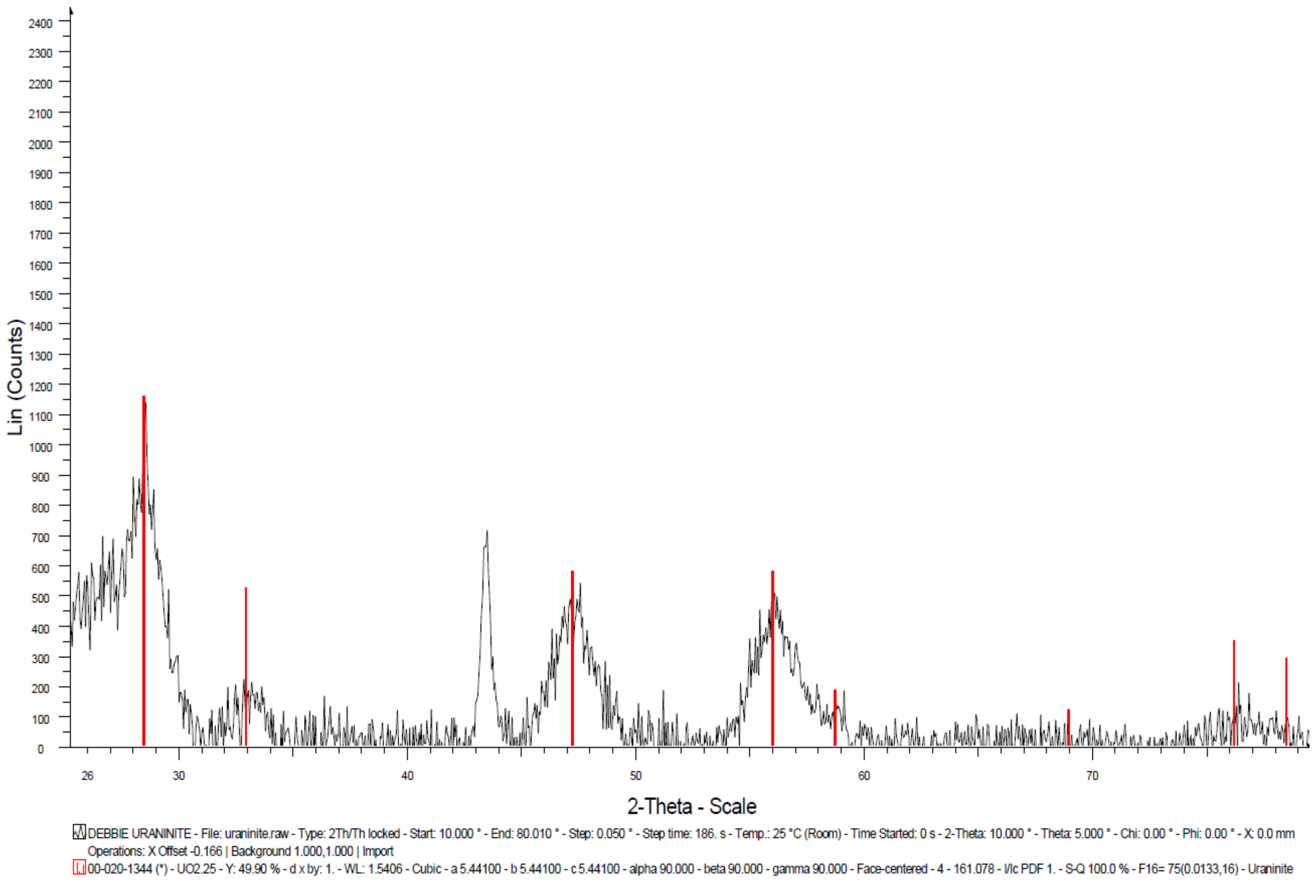

Fig. S12. PXRD pattern of black crystalline species formed during the bio-reduction of uranium. Black trace is the recorded pattern, red trace is calculated pattern.

Liquid Scintillation Experiments

For analysis of the samples using liquid scintillation counting, a 100 µl sample was mixed with scintillant (Scintisafe 3, Fisher Scientific) and 1 mL HCl prior to counting on a LSC (Quantulus, PerkinElmer) instrument. All samples were run in triplicate.

Table S2. Average concentration of soluble uranium at various points during the bio-reduction process.

Time (hrs)	Avg concentration of uranium kBq mL^{-1}
0	0.487
1	0.465
2	0.439
3	0.420
4	0.360
5	0.275
6	0.213
8	0.210
24	0.128

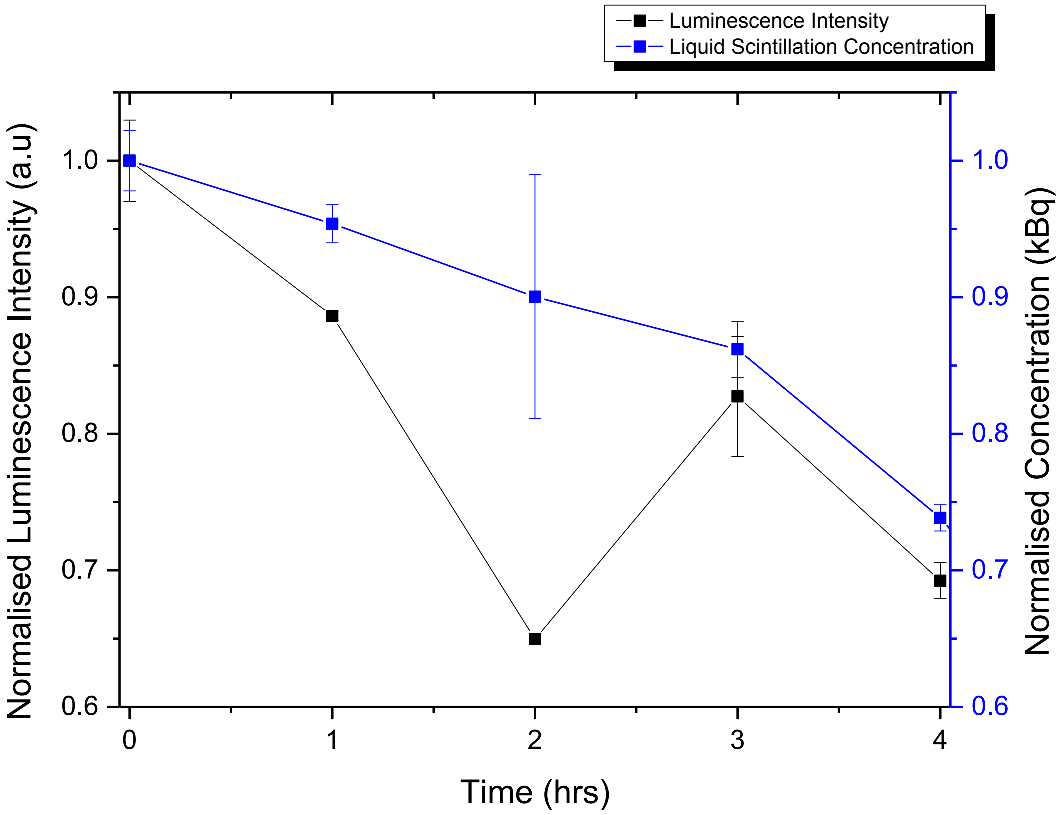

Fig. S13 Luminescence Intensity of the main 503 nm emission band plotted against liquid scintillation concentration values for the first 4 hours of the bio-reduction. Values normalised against reading taken at 0 hours.

Control Experiments

Experiments were conducted as described above using bacteria which were autoclaved at 125°C to confirm that the reduction of uranium was occurring solely due to enzymatic processes mediated by living bacteria and not due to any other (trace) reducing species. As Fig. S14 (below) shows, the concentration of uranium decreased only slightly across the 8 hour period indicating that the metabolically active bacteria play a vital role in the reduction of the uranium.

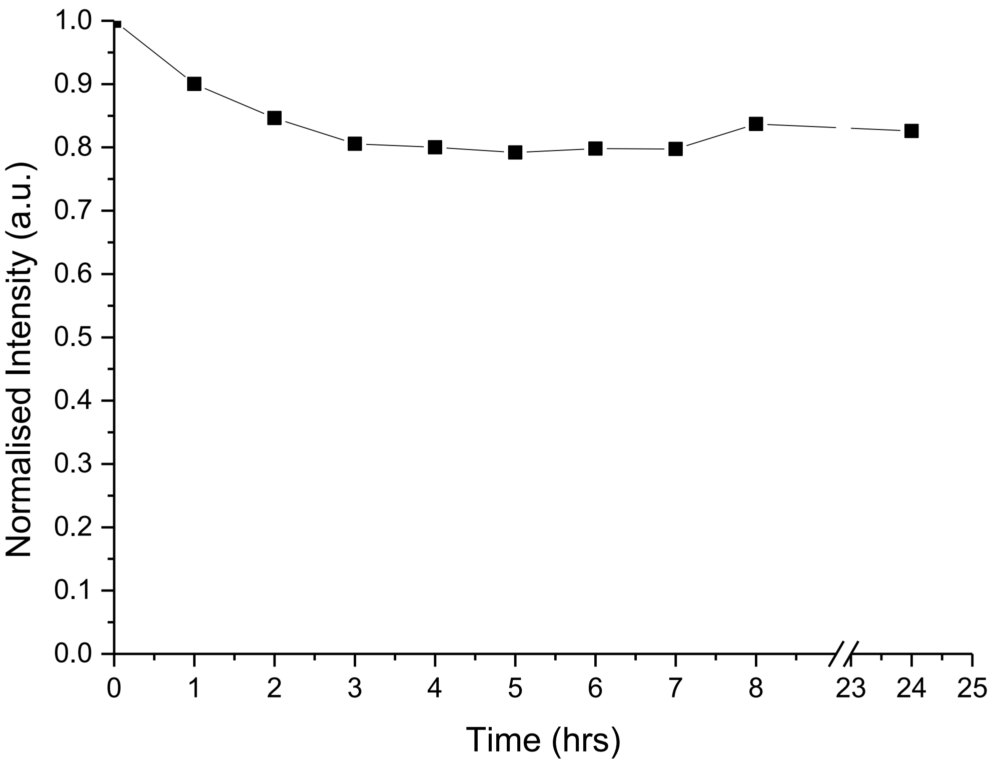

Fig. S14. Plot depicting luminescence intensity of uranium with autoclaved dead cells of *Geobacter sulfurreducens*; data normalised according to intensity at 0 hours.

Further control experiments were carried out using *Escherichia coli*, which was grown as described for *Geobacter sulfurreducens*. Samples with and without uranyl acetate were studied.

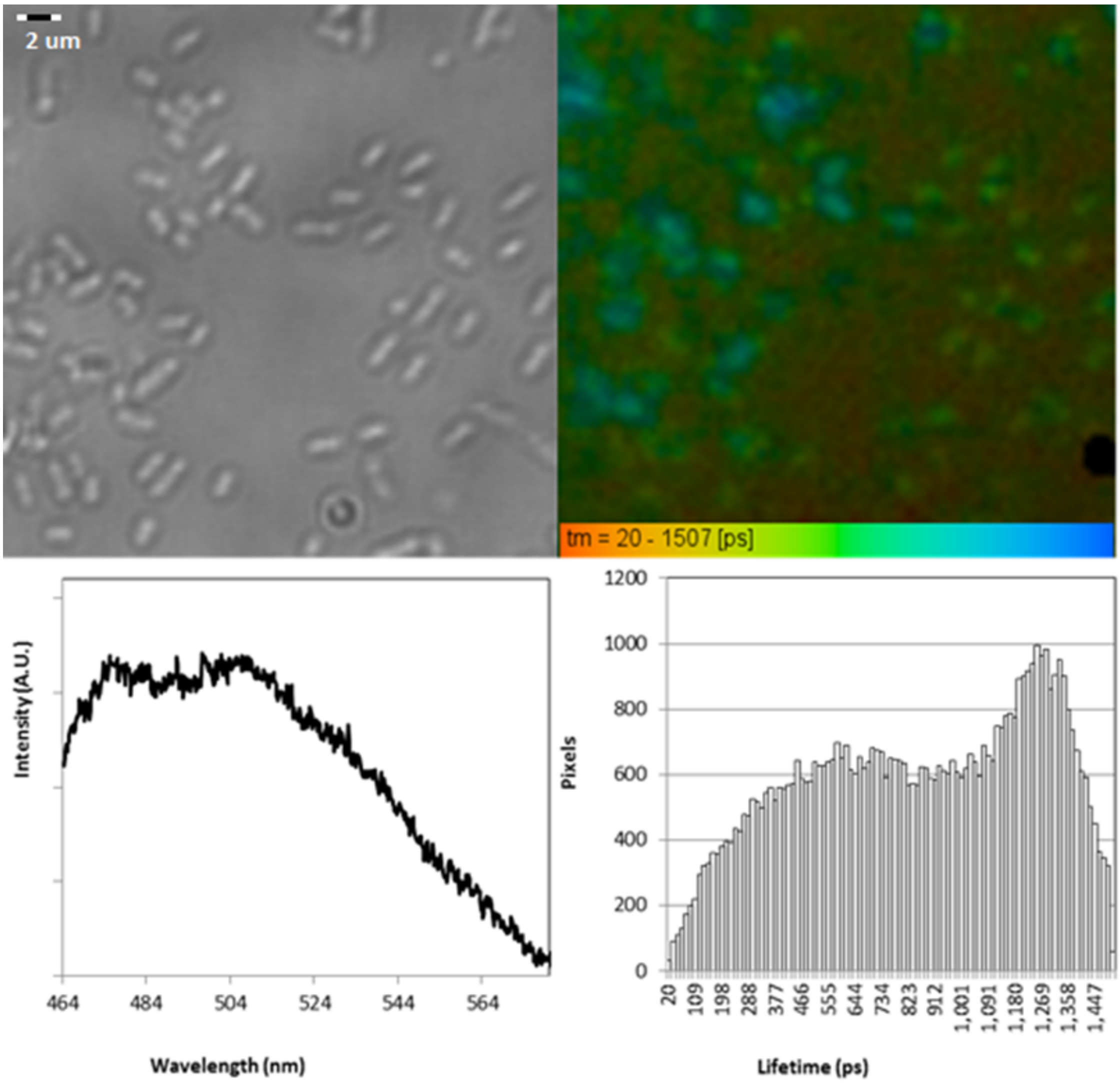

Fig. S15. Brightfield (top left) and FLIM (top right) microscopy images of *E. coli* in the absence of uranyl acetate (scale bar applies to both) as well as a representative spectrum taken from the surface of a cell (bottom left) and histogram showing fluorescence lifetime distribution (bottom right). All fluorescence results obtained using 405 nm excitation.

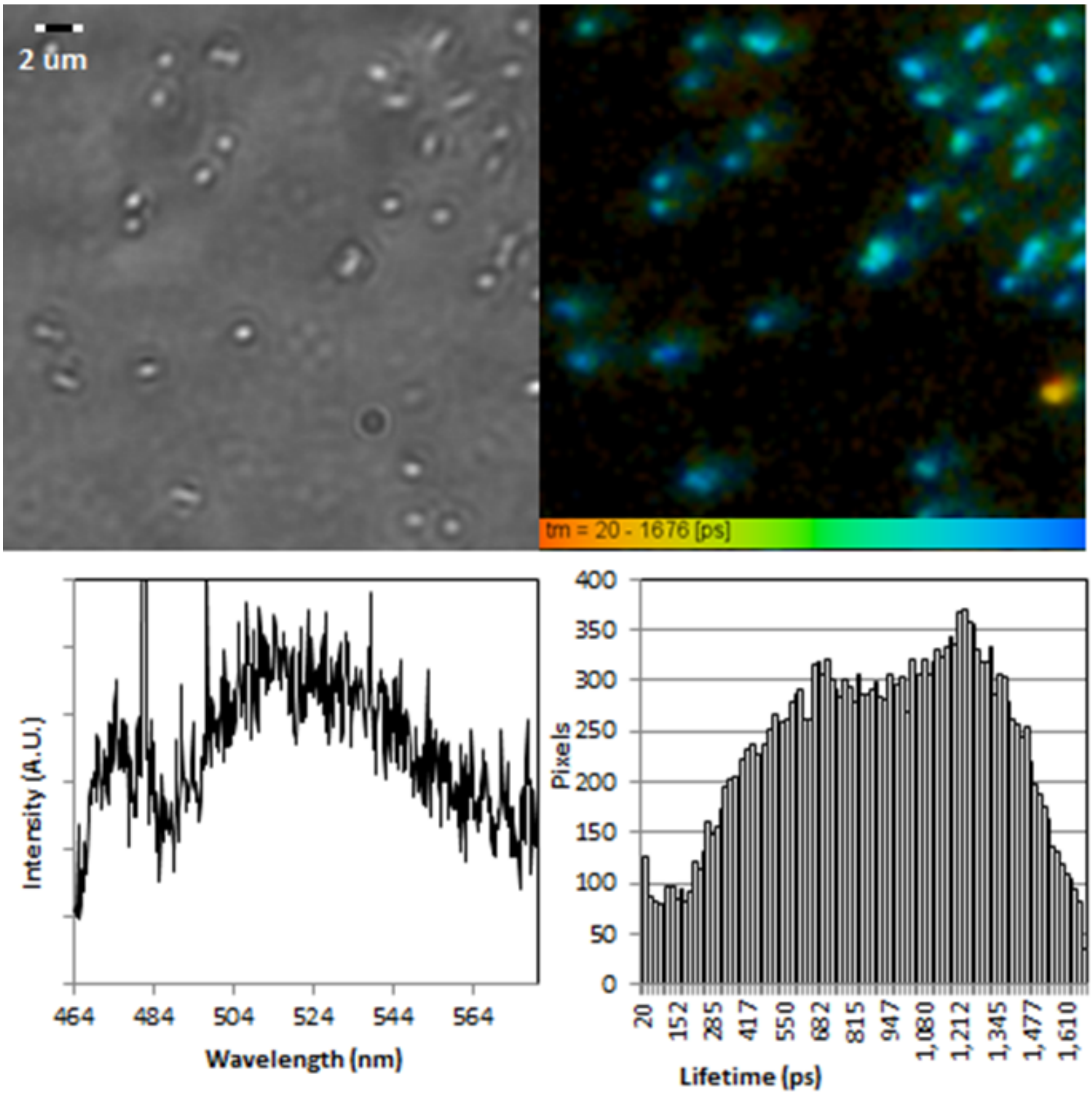

Fig. S16. Brightfield (top left) and FLIM (top right) microscopy images of *E. coli* in the presence of 5 mmol uranyl acetate (scale bar applies to both) as well as a representative spectrum taken from the surface of a cell (bottom left) and histogram showing fluorescence lifetime distribution (bottom right). All fluorescence results obtained using 405 nm excitation.

Additional FLIM/PLIM data

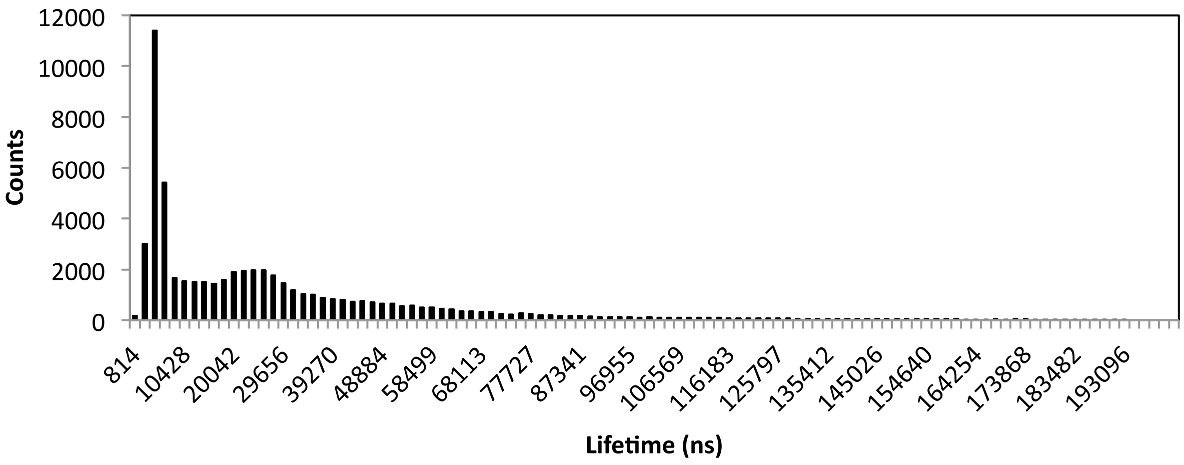

Fig. S17. Histogram showing full distribution of emission lifetime measurements in a PLIM experiment from one uranyl-containing extracellular feature following excitation at 405 nm.

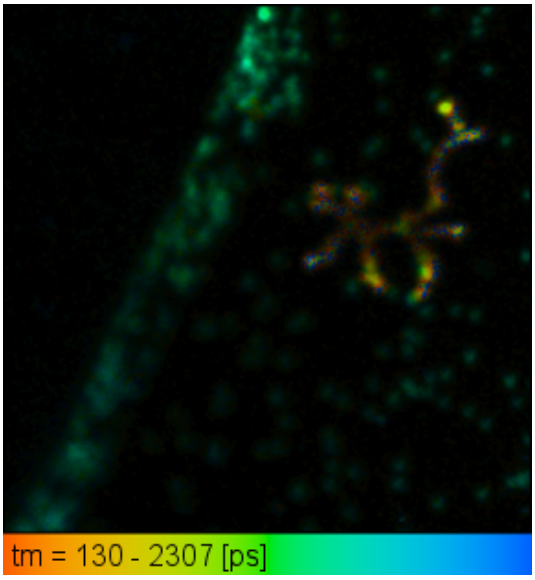

Fig. S18. FLIM image of *Geobacter Sulfurreducens* alongside one uranyl-containing extracellular feature following excitation at 405 nm.

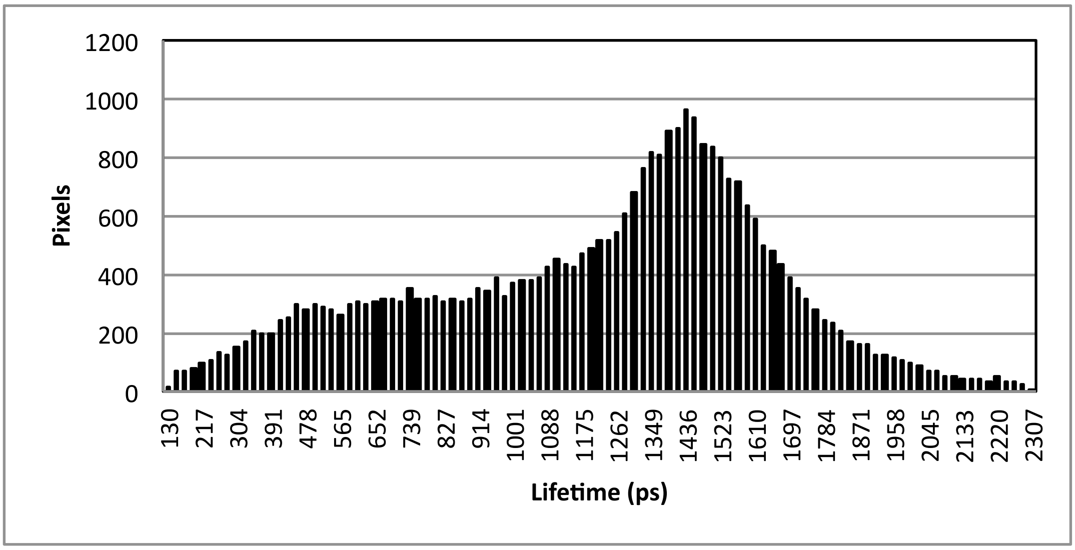

Fig. S19. Histogram showing full distribution of emission lifetime measurements from the FLIM image in Fig. S18 (405 nm excitation).
